# Systematic review and meta-analyses of suicidal outcomes following fictional portrayals of suicide and suicide attempt in entertainment media

**DOI:** 10.1016/j.eclinm.2021.100922

**Published:** 2021-06-04

**Authors:** Thomas Niederkrotenthaler, Stefanie Kirchner, Benedikt Till, Mark Sinyor, Ulrich S. Tran, Jane Pirkis, Matthew J. Spittal

**Affiliations:** a Unit Suicide Research & Mental Health Promotion, Department of Social and Preventive Medicine, Center for Public Health, Medical University of Vienna, Vienna, Austria; b Wiener Werkstaette for Suicide Research, Vienna, Austria; c Department of Psychiatry, Sunnybrook Health Sciences Centre, Toronto, Canada; d Department of Psychiatry, University of Toronto, Toronto, Canada; e School of Psychology, Department of Cognition, Emotion, and Methods in Psychology, University of Vienna, Vienna, Austria; f Centre for Mental Health, Melbourne School of Population and Global Health, The University of Melbourne, Melbourne, Australia

**Keywords:** Media, Suicide, Meta-analysis, Werther effect, Entertainment, Fiction

## Abstract

**Background:**

Guidelines to encourage responsible reporting of suicide in news media are a key component of suicide prevention strategies. Recent guidelines have been developed on portrayal of suicide in entertainment media although the relationship between these portrayals and subsequent suicidal behaviour has received considerably less attention in research.

**Methods:**

We conducted a systematic review and meta-analysis to examine the association between portrayals of suicide and suicide attempt in entertainment media and suicidal behaviour in the population. We searched PubMed, Scopus, Embase, PsycInfo, Web of Science and Google Scholar until April 20, 2021. We included studies adopting interrupted time series or single/multiple arm pre-post designs. Separate analyses were undertaken for studies of suicide and suicide attempts. We synthesized studies at moderate risk of bias and included studies at serious risk in a sensitivity analysis. Using a random-effects meta-analysis, we synthesized studies at moderate risk of bias and included studies at serious risk in a sensitivity analysis. Study registration: PROSPERO (CRD42020221333).

**Findings:**

Twelve studies met our inclusion criteria. Six studies were about suicide. Two of these were at moderate risk of bias and both examined the effects of the Netflix series *13 Reasons Why*. The pooled rate ratio (RR) for these studies was 1·18 (95% CI 1·09 to 1·27, *p*<0·001). Heterogeneity was low (I^2^ = 29%). Six studies focused on suicide attempts, and two of them were at moderate risk of bias. The pooled RR for these two studies was 1·33 (95% CI 0·84 to 2·09, *p* = 0·22). Heterogeneity was high (I^2^ = 92%). Enhanced funnel plots indicated likely publication bias for studies of suicide and possible bias for studies of attempted suicide.

**Interpretation:**

Portrayals of suicide in entertainment media may increase suicides and attempted suicide in the population. More studies that limit the potential sources of bias are needed to fully understand the circumstances under which fictional portrayals may influence suicidal behaviour.

**Funding:**

None.

**Research in context**

**Evidence before this study**

We undertook a systematic search of five databases (PubMed/Medline, Embase, PsychInfo, Scopus, Web of Science) up to April 20, 2021 and searched the reference list of all relevant primary studies for studies reporting on portrayals of suicide in entertainment media. We used the following search terms: ((suicid* OR self-harm*) AND (Werther OR Papageno OR copycat OR imitat* OR contagio* OR suggesti*) AND (media OR newspaper* OR print OR press OR radio* OR televis* OR film* OR movie OR book* OR documentar* OR internet OR cyber* OR web* OR music* OR song* OR play* OR theat*)). An additional cited-reference search was conducted for all relevant primary articles with Google Scholar. No language restriction was applied. We identified 12 individual studies and one meta-analysis, including four studies at moderate risk of bias and six studies at serious risk. The individual studies showed differing results – some showed no association between portrayals of suicide and suicide attempt in entertainment media and subsequent suicidal behaviour, whereas others showed a harmful effect, especially for the recent Netflix show 13 Reasons Why.

**Added value of this study**

Recent fictional portrayals of suicidal behaviour that applied an unambiguously harmful narrative focusing on suicide death appeared to be most consistently related to a relevant increase in suicides and attempts, but more studies of appropriate quality are needed.

**Implications of all the available evidence**

This study provides important evidence supporting the use of recommendations for how to portray suicide and suicide attempt in entertainment media in a way that is appropriate, sensitive and minimises the risk of harm. More studies of appropriate quality are needed to further elucidate the impact of particular narratives.

Alt-text: Unlabelled box

## 1 Introduction

Our recent meta-analysis about suicide portrayals in news and information media and its association with subsequent suicides showed that media reporting about celebrity suicide was associated with an increase of 8–18% in suicides in the population in the following 1–2 months [1]. The available evidence, however, is much less clear for portrayals in entertainment media but this issue has recently received considerable attention. The 2017 Netflix series *13 Reasons Why*, about the suicide of its fictional character, 17-year-old Hannah Baker, sparked strong criticism from mental health and suicide prevention organizations because it did not follow widely recognised media recommendations about how suicide should be portrayed in the media [2]. The concern was that the problematic narratives and messaging within the show could trigger additional youth suicides [3,4]. Specifically, concerns were based in the explicit imagery of a suicide method (cutting), the portrayal of help-seeking as being futile and counter-productive, and the notion that suicide might have a beneficial effect on others. Three subsequent studies found evidence that the series was indeed associated with a significant increase in suicides by young people in the United States [3,5] and Canada, [4] corroborating earlier clinical findings of increased presentations for self-harm amongst adolescents who had watched the series [3,4]. Sustained criticism from experts and these findings led the World Health Organization (WHO) and the US National Action Alliance for Suicide Prevention to develop specific media recommendations for the portrayal of suicide in entertainment media, [6,7] and Netflix responded by removing the controversial suicide scene from season one [8].

It remains unclear if portrayals of suicide and attempted suicide in entertainment media more generally are associated with subsequent suicidal behaviour. One previous meta-analysis found no evidence of imitative acts of suicide in populations exposed to suicide in entertainment media [9]. However, this meta-analysis combined very different study designs (e.g., experimental RCT studies and ecological time-series studies), used several different outcome variables (e.g., suicides, symptoms of depression), did not include several key studies, and did not provide a structured assessment of study quality. The author concluded that portrayals of suicide in entertainment media had no impact on suicidal behaviour, although this interpretation may not be correct given the substantial limitations of the study, and the evidence from excluded studies [10].

In the present study, we sought to update and summarise the available evidence about the relationship between portrayals of (attempted) suicide in entertainment media and suicide and suicide attempts in the population. We used a similar approach to our previous meta-analysis on suicide portrayals in news and information media, mentioned above [1].

## 2 Methods

Our meta-analysis was registered with PROSPERO (CRD42020221333) and no substantial modifications were made to the design of the study after registration. The study is reported using PRISMA guidelines.

### 2.1 Search strategy and selection criteria

We searched PubMed, Scopus, Embase, PsycInfo, and Web of Science on April 21, 2021 using the following search terms: ((suicid* OR self-harm*) AND (Werther OR Papageno OR copycat OR imitat* OR contagio* OR suggesti*) AND (media OR newspaper* OR print OR press OR radio* OR televis* OR film* OR movie OR book* OR documentar* OR internet OR cyber* OR web* OR music* OR song* OR play* OR theat*)). Titles, abstracts and keywords were searched from the date of inception of the respective database until April 20, 2021. No filters were applied to the searches. Additionally, we screened the reference lists of all retrieved full-text articles for any further articles and conducted a cited-reference search for all relevant primary articles using Google Scholar. Studies were included that (a) adopted an ecologic design comparing at least one time point before and one time point after suicide-related media portrayals; (b) included total deaths by suicide and/or suicide attempts as the outcome variable; and (c) were about fictional suicides or suicide attempts. Publications were excluded if they did not present primary or secondary data. If publications overlapped (i.e., data on the same portrayals in the same setting were reported in several articles; or the same study was reported in several publications), we included only one of these publications, selecting the publication with the following hierarchical approach: (a) had the highest quality (see below); and (b) included the largest number of portrayals (appendix, p3). The search and screening of articles for eligibility was done by SK and TN and discrepancies were discussed and resolved.

### 2.2 Data extraction

From each publication we extracted the type of outcome (suicide or suicide attempt), age group studied, and the follow-up time. If a specific suicide method was reported, we coded the method. For studies with suicide attempt as an outcome, because the majority focused on self-poisoning, we also extracted whether the study reported on total suicide attempts across all methods and/or self-poisoning specifically. Further, we abstracted the title of the broadcast or media product and broadcast network where the portrayal was released, the number of portrayals analysed, media (input) type (television series, television movie, streaming service), if the portrayals featured suicide or non-lethal suicidal behaviour (i.e., if the narrative was about a suicide, suicide attempts, or lives being saved), and we abstracted if there was a specific target audience of the portrayal analysed (unspecified, adults, teenagers/young adults). If publications reported suicides or suicide attempts in several demographic groups, we extracted data from the original publication's primary analysis. If publications reported on suicides or suicide attempts by specific suicide methods in addition to total suicides or suicide attempts, we extracted data about total suicides or suicide attempts. We obtained rate ratios and standard errors from each original study by one of the following methods (appendix, p5):

• Extracting a rate ratio and a standard error, 95% confidence interval, *t* value, or other estimate in order to calculate a standard error.
• Extracting the number of expected and observed suicides or suicide attempts and using this to calculate a rate ratio and standard error.
• Extracting the observed number of suicides or suicide attempts in the before and after exposure periods (along with the corresponding times) and calculating a rate ratio and standard errors.

In the case of studies led by two of us (TN and MS)[3,4], additional information was provided so we could recalculate rate ratios and standard errors (see below). Data abstraction was done by MJS who discussed the abstracted data with TN. No discrepancies emerged.

### 2.3 Measuring risk of bias

Risk of bias was assessed for each included study based on the Robins-I tool in an identical way to that used in our previous meta-analysis on reporting of suicide in news and information media [10]. We used a specific adaption for this study using six domains of bias: (a) bias due to confounding issues; (b) bias in classification of interventions; (c) bias due to preparatory phases; (d) bias due to missing data; (e) bias in measurement of the outcome; and (f) bias in selection of reported results.

Studies had an overall rating of “low risk of bias” if all domains were coded as “low risk”; “moderate risk” if one or more domains were coded as “moderate risk”; “serious risk” if at least one domain was assessed as “serious risk” but none as “critical”; and “critical” risk if any domain was coded as “critical risk”. As was the case in our previous meta-analysis, risk of bias due to confounding largely determined the total risk of bias in the present meta-analysis. Also, domain (c), risk of bias due to preparatory phases, was deemed relevant for several of the included studies. This domain codes bias that may result from, for example, promotion phases before movie releases, which would neither be clearly assignable to the pre-exposure nor to the post-exposure period. Most studies did not address preparatory phases, however, for the studies that two of us led, [3,4] we were able to re-analyse the original time series data including a dummy variable for a preparatory month before the actual movie release. For several other studies, the media portrayals were from soap operas rather than movies, which typically have a shorter promotion phase, and these studies were also coded as being at “moderate risk” in this domain even if they did not consider a preparatory phase. The full quality assessment is contained in the appendix, p8. Studies assessed as being at critical risk of bias were excluded. All risk of bias assessments were based on the data we abstracted. For example, if authors provided a re-analysis of their data, only that re-analysis was assessed, not the original study. The coding of risk of bias was done independently by TN and MJS, who discussed and resolved any discrepancies.

### 2.4 Data analysis

We describe the entertainment media portrayals of suicide and attempted suicide that were investigated in individual studies. We conducted a random-effects meta-analysis to estimate pooled rate ratios (RR) for the effects of portrayals of suicidal behaviour in entertainment media on suicides and suicide attempts in the population. Pooled RRs were calculated using REML estimation. We calculated pooled RRs for studies at up to moderate risk of bias. As a sensitivity analysis, we repeated the analysis including studies at serious risk of bias. Heterogeneity was assessed using the I^2^ statistic and Cochran's Q test. For I^2^, values around 25% indicated low heterogeneity, 50% moderate heterogeneity, and around 75% high heterogeneity [11]. We evaluated publication bias using contour-enhanced funnel plots [12]. All analyses were conducted by MJS and undertaken in Stata 16.1.

### 2.5 Role of the funding source

There was no funder for this study. All authors had full access to the data in the study. TN and MJS had final responsibility for the decision to submit for publication.

## 3 Results

**Fig. 1:**
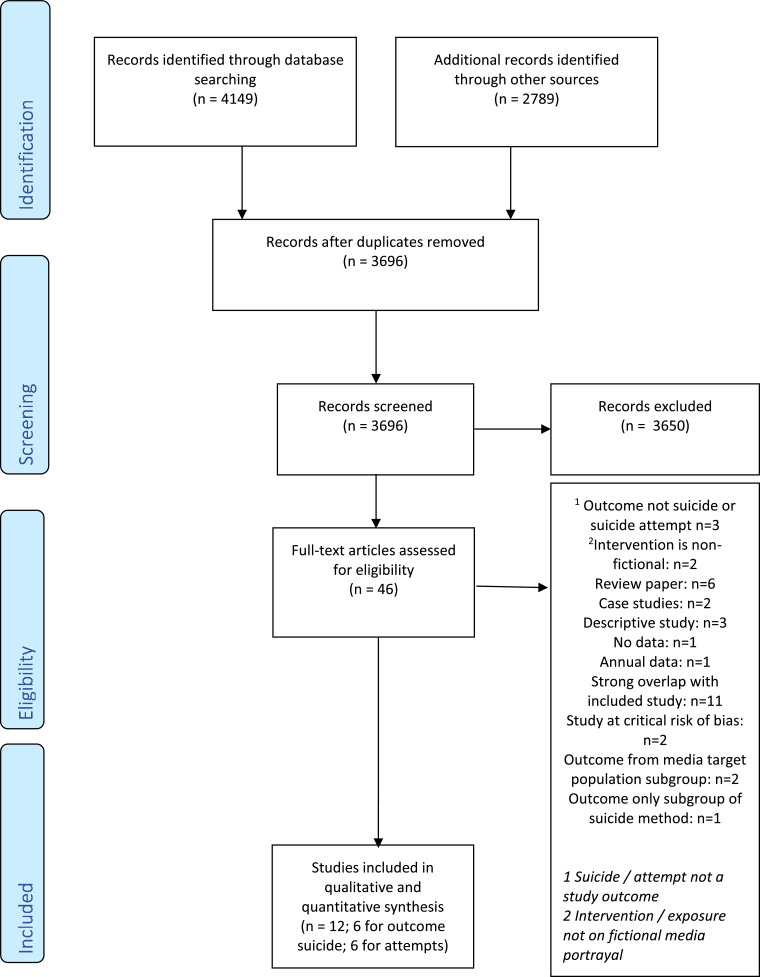
PRISMA flow diagram 3696 records identified by searching PubMed, Scopus, Embase, PsycInfo, and Web of Science were screened from inception to April 20 2021, for studies on fictional portrays of suicide and suicidal behaviour. In total, 12 studies were selected for qualitative and quantitative synthesis – 6 studies where suicide was the outcome and 6 where suicide attempts was the outcome.

We retrieved 6938 publications. 3696 remained after removing duplicates (Fig. 1). After screening the titles and abstracts, the full texts of 46 studies were assessed for eligibility and 33 excluded. The reasons for exclusion were because the outcome was not suicide or attempted suicide (3 studies), the media intervention was a non-fictional portrayal (2 studies), the article was a review study (6 studies) or a case study (2 studies), the study was descriptive only without sufficient data for abstraction (3 studies), no data was presented (1 study), data was collected annually and therefore not fine-grained enough for our analysis (1 study), the study had strong overlap with data presented in another study (11 studies), the portrayal targeted a specific population subgroup (2 studies) or data was collected from only a subgroup of a specific method (1 study). After quality assessment, we also excluded two studies at critical risk of bias. The remaining 12 studies were included in our review: 6 that examined changes in suicide counts following the portrayals of suicidal events in television or movies, and 6 that examined changes in suicide attempt counts (Table 1). Details about the risk of bias assessments is contained in the appendix, p8.

**Table 1:** Characteristics of the included studies.

First author and year	Country	Study period	Study length	Follow-up length	Outcome	Title of movie (network)	Number of media portrayals	Media type	Narrative about suicide versus suicide attempt	Target audience	Total risk of bias
**Suicide studies**											
Holding 1975	UK (Edinburgh)	1969–1972	150 weeks	4 to 14 weeks	Suicide by any method or death by undetermined intent	Befrienders (BBC)	11 episodes over 10 weeks	Television series	Mixed: One episode ends with death from suicide; a wide range of suicide-related behaviours is included	Unspecific	Serious
Kessler 1984	USA	Feb 1977- Nov 1977	88 days	4 days (day 0 to 3 after broadcast)	Suicide by any method	See Table 1, Kessler (1984)	15	Television series	Mixed: only 2 of 15 soaps feature fatal suicide attempts	Unspecific / adults	Serious
Berman 1988	USA	Dec 1984-Oct 1986	12 weeks	Following 2 weeks (some analyses with 4 weeks follow-up)	Suicide by any method	“A Reason to Live” (NBC, 1985); Surviving (ABC, 1985); “A Desperate Exit (ABC, 1986)	3	Television movies	Mixed: A Reason to Live features a suicide attempt	Teenagers	Serious
Schmidtke 1988	Germany	Jan 1976-Dec 1984	9 times 71 days (639 days) and 9 times 69 days (621 days)	70 days and 68 days	Suicide by rail suicide	Tod eines Schuelers [Death of a student] (ZDF, 1981)	1	Television series in 6 episodes	One specific suicide	Teenagers / young adults	Serious
Niederkrotenthaler 2019	USA	Jan 1999-Dec 2017	228 months	3 months (April to June 2017)	Suicide by any method	13 Reasons Why season 1 (Netflix, 2017)	1	Streaming service in 13 episodes	One specific suicide	Teenagers / young adults	Moderate
Sinyor 2019	Canada	Jan 2013–Dec 2017	60 months	9 months (April to December 2017)	Suicide by any method	13 Reasons Why season 1 (Netflix, 2017)	1	Streaming service in 13 episodes	One specific suicide	Teenagers / young adults	Moderate
**Suicide attempt studies**											
Cooper 2018	USA, Oklahoma Children's Hospital Medical centre	Jan 2012 – Oct 2017	70 months	3 months (April to June 2017)	Suicide attempts by any method: <19-year olds	13 Reasons Why season 1 (Netflix, 2017)	1	Streaming service in 13 episodes	One specific suicide	Teenagers / young adults	Moderate
Holding 1974	UK (Edinburgh)Regional Poisoning Treatment centre	1969–1972	72 weeks	4 to 14 weeks	Suicide attempts from self-poisoning	Befrienders (BBC)	11 episodes over 10 weeks	Television series	Mixed: one episode ends with death from suicide; several attempts	Unspecific	Serious
Simkin 1994	UK (Oxford), Oxford Monitoring System for Attempted Suicide	1990 Dec – 1993 Aug	36 weeks	3 weeks	Suicide attempts from paracetamol and non-paracetamol overdoses	Casualty (BBC)-Episode screened on July 16, 1993—and originally screened January 9, 1993	1	Television series	Unclear but likely a suicide: One near-fatal attempt from paracetamol poisoning with implication to die).	Unspecific (suicidal behaviour by teenage girl)	Serious
Platt 1987	63 hospitals throughout UK	Feb 1985 – March 1986	4 weeks	1 week	Suicide attempts from self-poisoning	Eastenders (BBC); episode screened on Feb 27, 1986 (‘Angie's overdose’)	1	Television series	Unclear outcome of a suicide attempt, possibly fatal	Unspecific	Serious
Hawton 1999	49 accident and emergency departments in UK	1996 Oct – Nov	6 weeks	3 weeks	Suicide attempts from self-poisoning	Casualty (BBC) Episode screened on Nov 2, 1996	1	Television series	Unclear outcome of a suicide attempt; Near-fatal with implication to die	Unspecific	Moderate
Gould 1986	6 hospitals; Greater New York Area, USA	Oct 1984 – Feb 1985	16 weeks (text says 25 weeks, but 16 weeks displayed in table)	2 weeks	Suicide attempts by any method	“A Reason to Live” (NBC, 1985); Surviving (ABC, 1985); and 2 other TV movies	2 use for meta-analysis (broadcast 1 and 2 removed due to overlapping periods)	Television movies	Mixed: A Reason to Live features a suicide attempt	Teenagers	Serious

Amongst studies that used suicide as their outcome, two studies were judged to be at moderate risk of bias. Both of these studies were about the series *13 Reasons Why* which was streamed in March 2017. One study, by Niederkrotenthaler and colleagues, [3] used US data; the other, conducted by Sinyor and colleagues, [4] used Canadian data. Both studies used interrupted time-series designs to quantify changes in the number of suicides amongst young people (the show's target audience), after adjustment for long-term trends, seasonality and autocorrelation. The remaining four studies did not meet the threshold for being at moderate risk of bias because of major methodological limitations (e.g., no adjustment for long-term suicide trends and no adjustment for potential confounders, appendix p8). Holding [13] examined changes in suicides in Edinburgh, Scotland after the 1972 broadcast of an 11-episode weekly television series, *The Befrienders*, which featured the role of The Samaritans as a suicide prevention service and included a variety of suicide-related behaviours including a suicide. Kessler and Stipp [14] examined the impact of 15 depictions of suicide or attempted suicide appearing in US television soap operas during 1977. Schmidtke and Häfner [15] examined changes in rail suicides following the broadcast of a 6-episode weekly series, originally in 1981 and again in 1982, focusing on a suicide by this method. After the character died in the first episode, the focus in all subsequent episodes was a search for reasons for the suicide. Berman [16] examined the effects of three made-for-television movies about suicide broadcast in the US in 1985 and 1986.

Two studies examining suicide attempts were identified as being at moderate risk of bias. Hawton and colleagues [19] compared the number of self-poisoning presentations to 49 UK emergency departments and psychiatric services in the 3 weeks before and after a 1996 episode of *Casualty* was broadcast. The episode featured a near-fatal suicide attempt by overdose. Cooper and colleagues [20] examined the effects of *13 Reasons Why* on presentations to a paediatric emergency department for attempted suicide in the 3 months following the release of the show. The four remaining studies about suicide attempts did not meet the threshold for being at moderate risk of bias. Holding [17] used the same fictional material as an earlier study [13] – the 1972 broadcast of *The Befrienders* series – to examine self-poisoning outcomes. Gould and Shaffer [18] examined the effects of four made-for-television movies on suicide attempts by any method. These were broadcast in 1984 and 1985. We were able to include data about two of the movies they studied, *A Reason to Live* and *Surviving* (the other two were broadcast close together in time, meaning the post-exposure period for one movie overlapped with the pre-exposure period of the subsequent movie). Platt [19] studied the effects of a specific episode within a long-running series. The show, *EastEnders,* featured an overdose by a young woman in a 1986 episode; Platt examined subsequent presentations for self-poisoning to major UK emergency departments. Simkin and colleagues [20] also studied a specific episode within the long-running series *Casualty*. The January 1993 episode (repeated in July 1993) featured a suicide attempt with an unclear outcome regarding the lethality of the attempt.

**Fig. 2:**
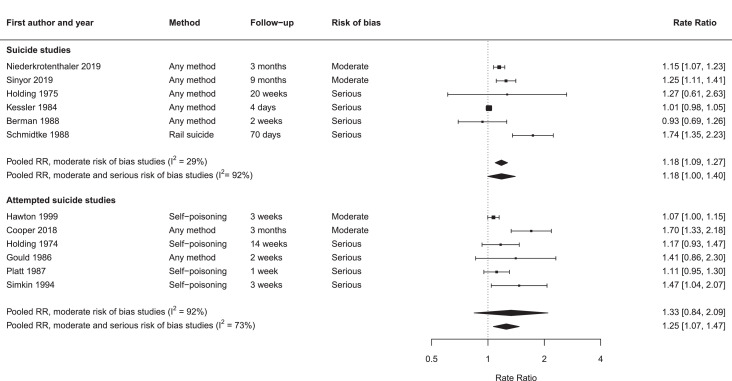
Forest plot of studies on entertainment media portrayals of suicidal behaviour Rate ratios and 95% confidence intervals for individual studies (squares and bars) and pooled rate ratio (and 95% confidence intervals) (diamonds).

For the two studies at moderate risk of bias in which suicide was the outcome of interest, the pooled RR was 1·18 (95% CI 1·09 to 1·27, *p* < 0·0001) (Fig. 2). The I^2^ value was 29%, indicating low heterogeneity. When the four studies at serious risk of bias were also included in the analysis, the pooled RR was 1·18 (95% CI 1·00 to 1·40, *p* = 0·054). The I^2^ value was 92%, indicating high heterogeneity.

For the two studies that examined suicide attempts as the outcome that were at moderate risk of bias, the pooled RR was 1·33 (95% CI 0·84 to 2·09, *p* = 0·22). I^2^ was 92%, indicating high heterogeneity. When the four studies at serious risk of bias were included in the analysis, the pooled RR was 1·25 (95% CI 1·07 to 1·47, *p* = 0·0055). The I^2^ value was 73%, indicating high heterogeneity.

**Fig. 3:**
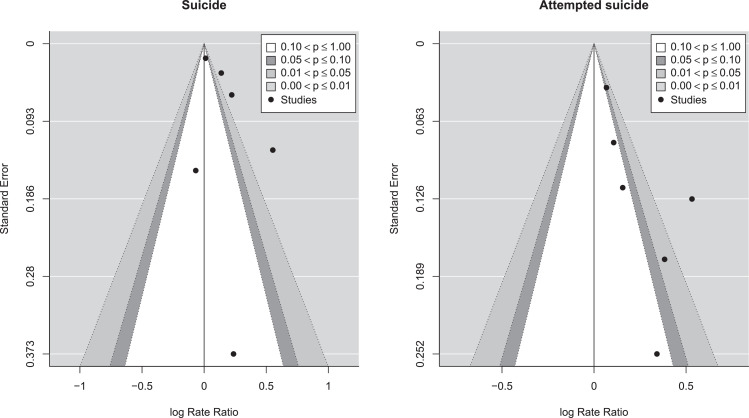
Contour-enhanced funnel plots Plots of the log rate ratio against its standard error (dark circles). The contours represent the significance level. Publication bias may be present if there is asymmetry around the null line of zero.

The contour-enhanced funnel plots (Fig. 3) suggested evidence of publication bias amongst the studies of suicide and possible publication bias amongst the studies of attempted suicide. For the studies of suicide, estimates from 4 of the 6 studies fell to the right of the null value. Three studies had estimates that were in the *p* < 1% zone. For studies of attempted suicide, all estimates fell to the right of the null line. One of the studies was in the *p* < 1% zone, two studies were in the 1% to 5% zone and the remaining 3 studies were in the >10% zone.

## 4 Discussion

In this meta-analysis, we found evidence that portrayals of suicide in entertainment media were associated with a 9% to 27% increase in suicides in the portrayal's target audience. The evidence was less clear with regard to suicide attempts. The two studies at moderate risk of bias showed either a weakly significant or a significant association between a portrayal of suicide and attempted suicide, but when analysed using a random-effects meta-analysis (which incorporates between-study heterogeneity into the calculation of the pooled estimate), we observed a non-significant association. These findings raise a number of important issues about the body of work and about the way suicidal behaviour is presented in entertainment media.

Our study highlights that, in spite of a substantial number of original studies in the topic area, the evidence on this topic is still limited. We believe there are several reasons for this. First, the literature is characterised by multiple publications that have analysed the same or similar exposure and outcome data [5,18,[21], [22], [23]]. We observed strong overlaps in US studies with regard to suicide. With regard to suicide attempts, there were strong overlaps in studies from the United Kingdom (appendix, p2–3).

Second, studies at moderate risk of bias are scarce and these were the studies that generally found an association with suicides and suicide attempts. Only two of six studies found evidence of an increase in suicides after suicide portrayals. These studies were both of moderate study quality, related to youth suicides and from the same fictional portrayal, *13 Reasons Why* [3,4]. Only one of the studies at serious risk of bias identified an association [15]. From the six studies on suicide attempts, [[17], [18], [19], [20],^24^,^25^] three studies identified a significant effect, whereas three found no association. But only two studies were judged to be at moderate risk of bias, showing effects that were consistent with the studies using suicide as the outcome.

A third important contributor to the limited evidence built so far appears to be that the actual characteristics of media portrayals differed strongly between and within studies, particularly for the studies judged at serious risk of bias. Heterogeneous narratives featured suicides, attempted suicides, and prevented suicidal behaviour sometimes combined into the same studies, [17,18] or suicidal behaviour with unclear outcome (Table 1) [[13], [14], [15], [16],^19^,^20^].

This point deserves further attention. There is good evidence from studies of news and information media that imitation effects are consistently observed for reports of suicide by celebrities [1]. Notably, this is also the most homogeneous media narrative (i.e., a highly identifiable and revered person dies by suicide). When that narrative changes, for example, as is in the case when the famous person who dies is considered a villain, no increases in suicides are observed [26,27]. If the story instead focuses on individuals who have managed to cope with adversity and master their suicidal crises, studies suggest that the effect might be reduced suicidal ideation [26], [27], [28], [29], [30]. In other words, these stories may have the opposite effect of stories depicting fatal or near-fatal suicidal behaviours.

This issue of including portrayals of different quality within the same original study are likely reflected in the present analysis. In *13 Reasons Why*, a show that unambiguously presents the narrative of suicide in an identifiable character, the overall effect clearly appeared to be harmful. The same was not observed for other, mainly older portrayals, which explored the impact of stories with very different content.

Studies on *13 Reasons Why* further differed from all other studies in that they were about a suicide portrayal that was streamed rather than traditionally broadcast. Streaming services had not been available in the past when people typically watched television in weekly episodes or went to the cinema at an appointed time. This may present additional risks to vulnerable viewers. With all content available on demand and at once, the reach of these portrayals is considerably greater than single episodes broadcast once a week. Even small effects have the potential to cause widespread harm if a large number of people can view shows at any time. This may also result in an under-estimation of actual effects for streaming services because there isn't a precisely defined broadcast date in which to evaluate outcomes. The present findings call for a renaissance of studies on media portrayals in entertainment media to investigate associations in the era of streaming services and online media.

Limitations of this study include our inability to determine causality due to the ecological nature of the original studies, which means that we do not know if individuals who died or made a suicide attempt after the broadcast were actually exposed to the media portrayal. In spite of the comprehensive search strategy, some non-English language studies may not have been indexed in the databases searched. Further, the only outcomes considered in this study were suicide and suicide attempts. These are the outcomes of greatest relevance to suicide prevention, but media portrayals might impact various other domains, including helpful outcomes (e.g., help-seeking behaviour) and harmful outcomes (e.g., stigmatization). Finally, all except three studies were carried out in the last century. In a rapidly changing media landscape and society, findings from more than two decades in the past might not directly translate to media effects found today.

In conclusion, our meta-analysis highlights the need for more high-quality studies in the area of the impact of portrayals of suicide and suicide attempt in entertainment media on suicidal behaviour. Inconsistent findings could reflect narrative differences, differences in media format (streaming vs. television), or poorer methodological quality of the earlier studies. Nevertheless, the synthesis of existing research is instructive. Similar to news media, stories about a protagonist with whom people strongly identify with and stories about a specific method of suicide, were consistently associated with harmful effects. These findings should be a major concern for filmmakers and prevention experts. Just as many journalists and editors are now alert to how to responsibly report on suicide in news and information media, [2] there is an onus on those who create and promote stories about suicide in entertainment media to take steps to prevent imitation effects. The entertainment media should adhere to existing guidelines around the safe portrayal of suicide, and during and after the release of the creative work, should work with experts to mitigate potential harms. One example of a collaboration between filmmakers and experts working well is the BBC's Channel 4′s 2012 youth drama *Hollyoaks*, where the writers and the Samaritans worked together to make changes to the script. Online support was also available to vulnerable audiences during the relevant episodes [31]. It is, however, important to note that no collaboration with mental health experts can ensure the safety of a specific portrayal if the standards outlined in existing guidance are not fully met. To ensure safety, portrayals of suicide methods should be avoided. Instead, showing that help is available and depicting characters who manage to cope with their suicidal thoughts and do not go on to make a suicide attempt is recommended. This study provides clear evidence supporting the use of recommendations to guide appropriate portrayal of suicide in entertainment media [6,7].

## Contributors

Study Concept and design: TN, MJS. Acquisition, analysis, or interpretation of data: All Authors; Drafting of the manuscript: TN, MJS; Critical revision of the manuscript for important intellectual content: All Authors; Statistical analysis: MJS. All authors had full access to all the data and accept responsibility to submit for publication.

## Data sharing

Data abstracted for this study are available from the authors upon reasonable request.

## Declaration of Competing Interest

Kirchner reports grants from Austrian Science Fund (FWF), project P30918-B27, during the conduct of the study. Dr. Sinyor reports personal fees from University of Toronto, Department of Psychiatry, personal fees from Sunnybrook Health Sciences Centre, Department of Psychiatry, grants from American Foundation for Suicide Prevention, grants from Canadian Institutes of Health Research, grants from Ontario Ministry of Research and Innovation, grants from Mental Health Research Canada, grants from Institute for the Advancements in Mental Health, grants from University of Toronto Miner's Lamp Innovation Fund, grants from Telus Canada, during the conduct of the study. Dr. Spittal is a recipient of an Australian Research Council Future Fellowship (FT180100075) funded by the Australian Government. Dr. Pirkis is funded by a National Health and Medical Research Council Investigator Grant (GNT1173126).

## Appendix Supplementary materials

Image, application 1
