# Supplementary material for: Systematic review and meta-analyses of suicidal outcomes following fictional portrayals of suicide and suicide attempt in entertainment media
Source: eClinicalMedicine. 2021 Jun 4;36:100922. doi: 10.1016/j.eclinm.2021.100922 (PMC8257930; doi:10.1016/j.eclinm.2021.100922)
Supplement: Supplementary file 1 [file mmc1.docx]

**Supplementary appendix to:**

**Systematic review and meta-analyses of suicidal outcomes following fictional portrayals of suicide and attempted suicide in entertainment media**

Thomas Niederkrotenthaler^1,2^, Stefanie Kirchner^1,2^, Benedikt Till^1,2^, Mark Sinyor^3^, Ulrich S. Tran^2,4^, Jane Pirkis^5^, Matthew J. Spittal^5^

^1^ Unit Suicide Research & Mental Health Promotion, Department of Social and Preventive Medicine, Center for Public Health, Medical University of Vienna

E-mails: [thomas.niederkrotenthaler@meduniwien.ac.at](mailto:thomas.niederkrotenthaler@meduniwien.ac.at); [benedikt.till@meduniwien.ac.at](mailto:benedikt.till@meduniwien.ac.at); [stefanie.kirchner@meduniwien.ac.at](mailto:stefanie.kirchner@meduniwien.ac.at)

^2^ Wiener Werkstaette for Suicide Research, Vienna, Austria

^3^ Department of Psychiatry, Sunnybrook Health Sciences Centre, Toronto, Canada.

Department of Psychiatry, University of Toronto, Toronto, Canada.

E-Mail: [mark.sinyor@sunnybrook.ca](mailto:mark.sinyor@sunnybrook.ca)

^4^ School of Psychology, Department of Cognition, Emotion, and Methods in Psychology, University of Vienna, Vienna, Austria. Email: [ulrich.tran@univie.ac.at](mailto:ulrich.tran@univie.ac.at)

^5^ Centre for Mental Health, Melbourne School of Population and Global Health, The University of Melbourne, Melbourne, Australia E-mails: [m.spittal@unimelb.edu.au](mailto:m.spittal@unimelb.edu.au);

[j.pirkis@unimelb.edu.au](mailto:j.pirkis@unimelb.edu.au)

**Table of Contents**

Outcome suicide: Overlapping studies from the United States and on 13 Reasons Why 3

Outcome suicide attempt from self-poisoning: overlapping studies of studies from the United Kingdom 4

Table S1: Detailed characteristics of the included studies with regards to study methods 5

Table S2: Risk of bias ratings of included studies based on Robins-I 9

# Outcome suicide: Overlapping studies from the United States and on 13 Reasons Why

We identified five studies from the United States that examined the effects of fictional movies of suicide in television films that were broadcast in 1985 and 1986 on suicide rates in young people (1-5). These studies had strong overlap in terms of the specific media portrayals included and time periods. Two of them drew methodological criticism due to overlapping exposure and control periods that were not accounted for in the analyses (1, 2, see 4). A replication of one of these studies (3) and another study in this study cluster (4) covered only a smaller region in the United States as compared to the fifth study in this study cluster (5). We therefore used the study which tested effects of these nationwide broadcasts in the largest region and with no apparent overlaps between intervention and control periods (5).

Two other studies both tested associations of fictional TV stories in the year 1977 with suicides in the United States (6, 7). One of them missed a few media portrayals (6) that were included in the second study, which was included in this review (7).

Finally, of the overall four studies on associations of *13 Reasons Why* with subsequent suicides on young people (8-11), three were focused on U.S. suicides and used the same database for the analysis (8-10). One study was on suicides in Ontario, Canada (11). Of the three U.S studies, we selected the study with the longest time period used for the analysis, and with the strongest modelling technique (8). The Canadian study was also included because there was no overlapping outcome data (11).

1. Gould M, Shaffer. The impact of suicide in television movies: evidence of imitation. New England Journal of Medicine. 1986; 315: 690-4.

2. Stack S. The impact of fictional television films on teenage suicide, 1984-85. Social Science Quarterly. 1990; 71(2): 392-99.

3. Gould MS, Shaffer D, Kleinman M. The impact of suicide in television movies: Replication and commentary. Suicide and Life‐Threatening Behavior. 1988; 18(1): 90-9.

4. Phillips DP, Paight DJ. The impact of televised movies about suicide. New England Journal of Medicine. 1987; 317(13): 809-11.

5. Berman AL. Fictional depiction of suicide in television films and imitation effects. The American Journal of Psychiatry. 1988; 148(8): 982-6.

6. Phillips DP. The impact of fictional television stories on U.S. adult fatalities: new evidence on the effect of the mass media on violence. American Journal of Sociology. 1982; 87(6): 1340-59.

7. Kessler RC, Stipp H. The impact of fictional television suicide stories on US fatalities: A replication. American Journal of Sociology. 1984; 90(1): 151-67.

8. Niederkrotenthaler T, Stack S, Till B, Sinyor M, Pirkis J, Garcia D, et al. Suicides in the United States after the release of 13 Reasons Why: Time series analysis. JAMA Psychiatry. 2019; 76: 933-40.

9. Bridge JA, Greenhouse JB, Ruch D, Stevens J, Ackerman J, Sheftall AH, et al. Association between the release of Netflix’s 13 Reasons Why and suicide rates in the United States: An interrupted time series analysis. Journal of the American Academy of Child & Adolescent Psychiatry. 2020; 59(2): 236-43.

10. Romer D. Reanalysis of the Bridge et al. study of suicide following release of 13 Reasons Why. PLOS one. 2020; 15(1): e0227545.

11. Sinyor M, Williams M, Tran US, Schaffer A, Kurdyak P, Pirkis J, et al. Suicides in Young People in Ontario Following the Release of “13 Reasons Why”. The Canadian Journal of Psychiatry. 2019; 64(11): 798-804.

# Outcome suicide attempt from self-poisoning: overlapping studies of studies from the United Kingdom

Several studies reported UK data on self-poisonings after an episode of *EastEnders*, a BBC soap opera that was aired on February 27 1986 and features Angie, a, “woman in her thirties” who makes a suicide attempt by poisoning. One of the studies used data from 63 UK hospitals [1], whereas four others had data from only one to three UK hospitals [2-5] that were potentially included in [1].

Two studies reported data about *Surviving*, an ABC production aired on February 10, 1985 that featured the suicides of a boy and girl. These studies reported about self-poisonings of adolescent in the same UK hospital [6-7]. We included neither because we judged them to be at critical risk of bias.

**References**

1. Platt S. The aftermath of Angie’s overdose: is soap (opera) damaging to your health? British Medical Journal. 1987, 294(6577), 954-7.
2. Williams JM, Lawton C, Ellis SJ, Walsh S, Reed J. Copycat suicide attempts. Lancet 1987, 2(8550), 102-3.
3. Sandler D, Connell P, Walsh K. Emotional crises imitating television. Lancet 1986, 1(8485), 956.
4. Fowler BP. Emotional crises imitating television. Lancet 1986, 1(8488), 1036-7.
5. Ellis S, Walsh S. Soap may seriously damage your health. Lancet 1986, 1(8482), 696.
6. Ostroff R, Behrends R, Lee K, Oliphant J.Adolescent suicides modeled after television movie. American Journal of Psychiatry 1985,142(8): 989.
7. Ostroff R, Boyd J. Television and suicide: Comment. New England Journal of Medicine 1987, 316(14): 876‐877

# Table S1: Detailed characteristics of the included studies with regards to study methods

| **First author and year** | **Study design** | **Data analysis** | **Method to control for time trends and seasonality** | **Confounders measured** | **Outcome data source** | **Number of pre-exposure time points** | **Number of post-exposure time points** | **Estimate** | **How estimate used in meta-analysis was derived*** | **Extracted data** |
| --- | --- | --- | --- | --- | --- | --- | --- | --- | --- | --- |
| Berman 1988 | Multiple arm pre-post comparison | Comparison of suicides in the pre- and post-exposure periods | None | None | Medical examiners’ offices in 20 US states | 2 weeks | 2 weeks | Unadjusted | Page 984, observed and expected number of suicides for people aged ≤19 years pre-vs. post exposure were extracted and converted to a log RR (with standard error) | Observed = 43 suicides, expected = 46 suicides. Log RR = -0.067 (0.152) |
| Cooper 2018 | Interrupted time series analysis | ARMA model of hospital admissions for attempted suicide by any method | Logged outcome variable, moving average and autoregressive terms, seasonal terms and 3^rd^ order polynomial terms | None | Hospital admission records from a single hospital in Oklahoma, US. | 65 months | 5 months | Unadjusted | Figure 1, observed and expected number of attempts for April, May and June 2017 extracted and converted to a log RR (with standard error) | Observed = 63 suicides, expected = 37 suicides. Log RR = 0.532 (0.126) |
| Gould 1986 | Multiple arm pre-post comparison | Comparison of suicides and attempted suicides by any method in the pre- and post-exposure periods | None | None | Data from a psychological and autopsy study conducted by Youth Suicide Research Unit at Columbia University (9 regions/counties) | 2 weeks | 2 weeks | Unadjusted | Attempted suicides: Reanalysis of data in Table 1, all attempts for broadcast II and IV pre-vs. post exposure to calculate RR (with standard error) | Broadcast 3, 10 attempts in the pre-exposure period and 19 attempts in the post-exposure period. Broadcast 4, 17 attempts in the pre-exposure period and 19 attempts in the post-exposure period. Log RR = 0.342 (0.252). |
| Hawton 1999 | Multiple arm pre-post comparison | Comparison of attempted suicides by self-poisoning in the pre- and post-exposure periods | None | Age, sex, choice of substance and centre | 49 accident and emergency departments and psychiatric services in the UK | 3 weeks | 3 weeks | Adjusted | Table 2, extracted RR for all overdoses, averaged over the three weeks post-exposure and the 95% CI. Converted to a log RR with standard error. | Log RR = 0.068 (0.036). |
| Holding 1974 | Multiple arm pre-post comparison | Comparison of attempted suicides by self-poisoning in the pre- and post-exposure periods | None | None | Admissions to the Regional Poisoning Treatment Centre, Edinburgh | 4 weeks | 14 weeks | Unadjusted | Reanalysis of data in Table II to calculate RR and its standard error | Log RR = 0.156 (0.117) |
| Holding 1975 | Multiple arm pre-post comparison | Comparison of suicides and deaths of undetermined in the pre- and post-exposure periods | None | None | Suicides and death of undetermined intent in Edinburgh extracted from the Registrar General’s Office in Scotland | 10 weeks | 20 weeks | Unadjusted | Reanalysis of data in Table II to calculate RR and its standard error | Log RR = 0.237 (0.373). |
| Kessler 1984 | Multiple arm pre-post comparison | Comparison of suicides in the pre- and post-exposure periods | None | None | US National Center for Health Statistics’ Mortality Detail file, 1977 | 3 days | 3 days | Unadjusted | Table 2, total experimental and control suicides (observed vs. expected) were extracted and converted to a RR (with standard error). | Observed = 3264 suicides, expected = 3225 suicides. Log RR = 0.012 (0.018). |
| Niederkrotenthaler 2019 | Interrupted time series analysis | ARIMA model of suicides | Detrending (for monthly and yearly trends) and inclusion of moving average terms (for month and year) | None | US Centers for Disease Control and Prevention WONDER database | 219 months | 3 months | Adjusted | Reanalysis of the data by the study authors to calculate observed and expected number of suicides. Converted to a RR (with standard error) | Observed = 804 suicides, expected = 700 suicides. Log RR = 0.138 (0.035). |
| Platt 1987 | Multiple arm pre-post comparison | Comparison of attempted suicide by self-poisoning in the pre- and post-exposure periods | None | None | 63 responding hospitals with large accident and emergency departments in Britain with populations ≥ 60,000 | 1 week | 1 week | Unadjusted | Table 1, whole sample, data extracted for control and experimental weeks in 1985 and 1986 and reanalysed to calculate RR (and its standard error) | 1985: 600 overdoses in the pre-exposure period and 613 in the post-exposure period. 1986: 599 overdoses in the pre-exposure period and 681 in the post-exposure period. Log RR = 0.107 (0.080). |
| Schmidtke 1988 | Single arm comparison | Comparison of rail suicide in exposure period with the same period in pre-exposure years and post-exposure years | None | None | Data from 9 regional rail offices in Germany (representing the entire rail network) | n/a | 70 days | Unadjusted | Figure 2, number of male and female suicides for people aged 15-29 years extracted. Reanalysis of data to calculate RR for 1981 (and its standard error) | Log RR = 0.553 (0.128). |
| Simkin 1994 | Multiple arm pre-post comparison | Comparison of attempted suicides by self-poisoning in the pre- and post-exposure periods | None | None | Oxford Monitoring System for Attempted Suicide | 3 weeks | 3 weeks | Unadjusted | Table 1, data extracted for poisoning by paracetamol and other drugs and reanalysed to calculate RR (and its standard error) | Control period, 220 overdoses in the pre-exposure period and 184 in the post-exposure period. Screening period, 88 overdoses in the pre-exposure period and 108 in the post-exposure period. Log RR = 0.383 (0.175). |
| Sinyor 2019 | Interrupted time series analysis | ARIMA model of suicides | Detrending (for monthly trends) and inclusion of a moving average term | None | Office of the Chief Coroner of Ontario | 50 months | 9 months | Adjusted | Reanalysis of the data by the study authors to calculate observed and expected number of suicides. Converted to a RR (with standard error) | Observed = 264 suicides, expected = 211 suicides. Log RR = 0.222 (0.062) |

*Table number refers to the specific table published in the original study

# Table S2: Risk of bias ratings of included studies based on Robins-I

| **First author and year** | **Q1-a** | **Q1-b** | **Q1-c** | **Q1-d** | **Q1-total** | **Q2** | **Q3** | **Q4** | **Q5** | **Q6-a** | **Q6-b** | **Q6-c** | **Q6-total** | **Total risk** |
| --- | --- | --- | --- | --- | --- | --- | --- | --- | --- | --- | --- | --- | --- | --- |
| Berman 1988 | No | Yes | Yes | Yes | Serious | Low | Serious | Low | Low | No | No | No | Moderate | Serious |
| Cooper 2018 | No | No | No | Yes | Moderate | Low | Moderate | Low | Low | No | No | No | Moderate | Moderate |
| Gould 1986 | No | Yes | No | Yes | Serious | Low | Serious | Low | Low | No | No | No | Moderate | Serious |
| Hawton 1999 | No | No | No | Yes | Moderate | Low | Moderate | Low | Low | No | No | No | Moderate | Moderate |
| Holding 1974 | No | Yes | No | Yes | Serious | Low | Serious | Low | Low | No | No | No | Moderate | Serious |
| Holding 1975 | No | Yes | No | Yes | Serious | Low | Serious | Low | Low | No | No | No | Moderate | Serious |
| Kessler 1986 | No | Yes | No | Yes | Serious | Low | Moderate | Low | Low | No | No | No | Moderate | Serious |
| Niederkrotenthaler 2019 | No | No | No | Yes | Moderate | Low | Moderate | Low | Low | No | No | No | Moderate | Moderate |
| Platt 1987 | No | Yes | No | Yes | Serious | Low | Moderate | Low | Low | No | No | No | Moderate | Serious |
| Schmidtke 1988 | No | Yes | No | Yes | Serious | Low | Moderate | Low | Low | No | No | No | Moderate | Serious |
| Simkin 1974 | No | Yes | No | Yes | Serious | Low | Moderate | Low | Low | No | No | No | Moderate | Serious |
| Sinyor 2019 | No | No | No | Yes | Moderate | Low | Moderate | Low | Low | No | No | No | Moderate | Moderate |

Abbreviations: Q1-a = Risk of bias due to confounding A: Sufficient pre-intervention (i.e., pre-release) time-points measured; Q1-b = Risk of bias due to confounding B: appropriate

analysis method that accounts for time trends; Q1-c = Risk of bias due to confounding C: Seasonality; Q1-d = Risk of bias due to confounding D: Confounders

measured and controlled for; Q1-total = Risk of bias due to confounding; Q2 = Risk of bias in classification of studies; Q3 = Risk of bias due to deviations from

preparatory phases; Q4 = Risk of bias due to missing data; Q5 = Risk of bias in measurement of the outcome; Q6-a = Risk of bias in selection of results: selection

of time point within series; Q6-b = Risk of bias in selection of results: selection of method from several conducted methods; Q6-c = Risk of bias in selection

of results: Selection of results because of interest, but more results available; Q6-total = Risk of bias in selection of results.

*For fictional media, preparatory phases can arise from the fact that they are promoted before the actual release, and therefore exposure to some of the content occurs before the release.

Risk of bias from preparatory phases was defined as *serious* if it was not accounted for and the broadcast was about a movie rather than a series or if the follow-up period of the study was

less than week, and *moderate* if the broadcast was from a series / soap opera with follow-up of at least one week. The bases for this definition was that promotion phases before releases

are typically stronger for movies than for series, and series are typically promoted only for a few days before their release (if they, e.g., screen every week). That means that promotion of series

mainly has an impact if the comparison period before the release is short / a few days rather than weeks.
